# Reduced central sympathetic activity in Parkinson's disease

**DOI:** 10.1002/brb3.1463

**Published:** 2019-11-05

**Authors:** Heidrun H. Krämer, Gothje Lautenschläger, Michael de Azevedo, Kathrin Doppler, Anne Schänzer, Christoph Best, Wolfgang H. Oertel, Iris Reuter, Claudia Sommer, Frank Birklein

**Affiliations:** ^1^ Department of Neurology Justus‐Liebig‐University Giessen Germany; ^2^ Department of Neurology University Hospital Würzburg Würzburg Germany; ^3^ Institute of Neuropathology Justus‐Liebig‐University Giessen Germany; ^4^ Department of Neurology Philipps‐University Marburg Germany; ^5^ Department of Neurology University Medical Center Johannes Gutenberg‐University Mainz Germany

**Keywords:** autonomic failure, MSNA, Parkinson's disease, phosphorylated α‐synuclein deposits

## Abstract

**Objective:**

With a combination of different sympathetic tests, we aimed to elucidate whether impairment of sympathetic function in Parkinson's disease (PD) is the consequence of a central or peripheral efferent dysfunction.

**Methods:**

Thirty‐five patients with early‐to‐intermediate PD (median age: 63 years; IQR: 57–67 years; disease duration 1–9 years, 15 women) and 20 age‐ and sex‐matched healthy controls (median age: 64.5 years; IQR: 58–68 years; 10 women) were recruited. Autonomic testing was performed in two subgroups and included the assessment of resting cardiovascular parameters, postprandial hypotension (PPH), orthostatic hypotension (OH), and vasoconstriction induced by intradermal microdialysis with different concentrations of norepinephrine (NE; 10^–5^; 10^–6^; 10^–7^; 10^–8^) and by cold through forehead cooling. We also used sympathetic multiunit microneurography (muscle sympathetic nerve activity; MSNA; burst frequency (BF): bursts per minute; burst incidence (BI): bursts per 100 heart beats) and evaluated the presence of phosphorylated α‐synuclein deposits in skin innervation in biopsies from the thighs by immunohistohemistry.

**Results:**

Diastolic blood pressure was higher in the PD group at rest (*p* < .001) and during OH (*F* = 6.533; *p* = .022). Vasoconstriction induced by NE microdialysis and cold was unchanged in PD patients. MSNA was lower in PD patients than in controls (BF: *p* = .001; BI: *p* = .025). Phosphorylated α‐synuclein deposits could be found only in PD patients.

**Conclusion:**

We did not find indications for peripheral sympathetic nerve fiber dysfunction or adrenoreceptor sensitivity changes. The decreased MSNA argues in favor of central sympathetic impairment.

## Graphical abstract

With a variety of autonomic tests, we aimed to further elucidate the pathophysiology of autonomic impairment in Parkinson's disease. Since MSNA is reduced, our data favor a central origin of autonomic disturbances. However, since peripheral impairment of, for example, QSART in PD is known, combined central and peripheral mechanisms seem likely.

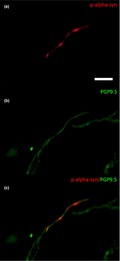

## 1 INTRODUCTION

In Parkinson's disease (PD), autonomic disturbances sometimes precede the typical motor triad, indicate progress with disease duration (Guo et al., 2013) and have been proposed as predictive markers (Palma & Kaufmann, 2014). The list of autonomic deficits in PD includes gastrointestinal, urinary tract, sexual, and sweating disturbances, drooling, and cardiovascular symptoms (Khoo et al., 2013), in particular orthostatic intolerance and postprandial hypotension (Sharabi, Imrich, Holmes, Pechnik, & Goldstein, 2008). An impaired baroreflex function in PD suggests both sympathetic and parasympathetic failure (Umehara, Toyoda, & Oka, 2014); scintigraphy (meta‐iodobenzylguanidine; MIBG) indicates impaired peripheral cardiac sympathetic transmitter reuptake (Takatsu et al., 2000).

Typical for PD is the reduction of epidermal small sensory and dermal autonomic fibers (Donadio et al., 2014). Phosphorylated α‐synuclein (P‐α‐synuclein) deposits could be identified in the skin (Donadio et al., 2014; Doppler et al., 2014). Recent studies showed negative correlations between α‐synuclein ratios and adrenergic function of autonomic fibers (Wang, Gibbons, Lafo, & Freeman, 2013) as well as between the amount of P‐α‐synuclein and small fiber degeneration (Donadio et al., 2014). In mice, the presence of these deposits is associated with a reduction of synaptic proteins, progressive deficiency in neuronal connectivity as well as excitability and finally neuronal death (Volpicelli‐Daley et al., 2011). Their potential clinical relevance for sympathetic denervation is indicated by their abundance in patients with pure autonomic failure (Donadio et al., 2013). It was only recently shown that in PD with orthostatic hypotension (OH) higher levels of P‐α‐synuclein deposits can be detected in autonomic nerve fibers in the skin compared to PD patients without OH (Donadio et al., 2018). However, it remained unclear whether these α‐synuclein deposits are associated with further clinically relevant autonomic deficits in PD.

We aimed to explore whether early‐to‐intermediate PD patients have an impairment of autonomic function, and if so, whether it would be due to peripheral mechanisms such as sympathetic nerve fiber degeneration and altered adrenoreceptor responsiveness, or whether such impairment rather would reflect central sympathetic disturbance. Therefore, we applied a combination of sympathetic tests.

## 2 MATERIAL & METHODS

### 2.1 Patients and controls

Thirty‐five patients with early‐to‐intermediate PD (median age: 63 years; IQR: 57–67 years; disease duration 1–9 years, 15 women) and age‐ and sex‐matched 20 healthy controls (median age: 64.5 years; IQR: 58–68 years; 10 women) were recruited. “Early‐to‐intermediate PD” was defined as Hoehn and Yahr (H&Y) (Hoehn & Yahr, 1967) scale I through III without adverse effects of treatment (Koller, 2002). PD patients taking antihypertensive drugs, having a medical history of hypertension or suffering from other diseases potentially causing autonomic failure were excluded. All patients were nonsmoker and had no history of alcohol or drug abuse. Six of the included patients suffered from hyperlipidemia and one from diabetes mellitus type II. Diagnosis of PD was made according to the United Kingdom Parkinson's disease Society Brain Bank criteria (Hughes, Daniel, Ben‐Shlomo, & Lees, 2002). A detailed medical history was recorded from all patients. Complete neurological examination was performed and disease staging was achieved using the H&Y scale (Hoehn & Yahr, 1967). Motor symptoms were quantified using the Unified Parkinson's disease scale part III (URPDS III) (Goetz et al., 2008). The median of the L‐dopa equivalent dose was 205 (IQR: 100–650). Nonmotor and autonomic symptoms were evaluated with the German version of the nonmotor symptom scale (NMSS). All patients reported autonomic symptoms (NMSS; median: 32; IQR: 13–67) (Storch et al., 2010). In all controls, the medical history and clinical neurological examination remained unremarkable. Subjects taking antihypertensive drugs, having a medical history of hypertension or suffering from other diseases potentially causing autonomic failure were excluded. All controls were nonsmoker and had no history of alcohol or drug abuse. Three of the included patients suffered from hyperlipidemia and none from diabetes mellitus type II.

All subjects underwent testing in a quiet, temperature‐, and humidity‐controlled laboratory. All participants gave their informed written consent, and all experiments were conducted in compliance with the latest revision of the Declaration of Helsinki. The study was approved by the ethics committee of the Rhineland‐Palatinate medical association as well as local ethics committee of the medical faculty of the Justus‐Liebig‐University, Giessen, Germany (161/11).

Due to the extensive protocol of the study, not all tests were performed in all participants. The patients were recruited from the outpatient clinics of the Neurological departments of the University Medical Center Mainz, Justus‐Liebig‐University Giessen and Philipps‐University, Marburg. The patients were divided into two groups and were investigated in two centers (Mainz and Giessen):

Patient group 1:20 patients suffering from early‐to‐intermediate stage idiopathic PD (median age: 65.5 (IQR 54.75–70 years), 13 women; H&Y 1: *n* = 2; H&Y 2: *n* = 14; H&Y 3: *n* = 4; URPDS III: median 10.0 (IQR: 7.0–25.0); disease duration: median 4 years (IQR 2–7 years); 10 age‐ and sex‐matched healthy subjects (median age: 68; IQR 66.25–74 years); 6 women) underwent the following investigations: (a) resting blood pressure and heart rate; (b) testing of postprandial hypotension; (c) analysis of vasoconstriction induced by intradermal microdialysis with norepinephrine, and (d) forehead cooling.

Patient group 2:15 patients suffering from early‐to‐intermediate stage idiopathic PD (median age: 62 years (IQR: 57.0–65 years); 6 women; H&Y 1: *n* = 6; H&Y 2: *n* = 7 H&Y 3: *n* = 2; URPDS III: median: 7.0 (IQR: 3.8–16.0); disease duration: median 3 years (IQR: 2–5 years)); 10 age‐ and sex‐matched healthy controls (median age: 58 years (IQR 57–61.25 years); 6 women) underwent the following investigations: (a) resting blood pressure and heart rate; (b) testing of orthostatic hypotension; (c) skin biopsy; and (d) assessment of muscle sympathetic nerve activity (MSNA).

There were no differences regarding the parameters assessed in the study: H & Y, heart rate and blood pressure. However, we cannot rule out that the two patient groups differed in other aspects of PD that we did not assess.

### 2.2 Cardiovascular parameters

To assess cardiovascular parameters at rest, blood pressure (systolic blood pressure; SBP; diastolic blood pressure; DBP), and heart rate (HR, bpm) were measured with a digital sphygmomanometer (WEPA) after 15 min resting in the supine position.

### 2.3 Postprandial hypotension (PPH)

Postprandial hypotension is defined as a decrease in SBP of at least 20 mmHg within 1 hr after drinking 75 g of glucose (Umehara et al., 2014). After overnight fasting (except for water), the study started at 9:00 a.m. After 20 min of resting (baseline) in supine position, the subjects drank 75 g of glucose water (calorie content: 300 kcal) and remained lying for 60 min. Blood pressure and heart rate were measured at baseline and after 15, 30, 45, and 60 min. The maximum drop in SBP (⊿SBP_max) was calculated. Plasma glucose (PG) concentrations were measured at baseline (PG_baseline_) and after 30, 60, and 90 min using the hexokinase method. The differences between PG_baseline_ and each of the time points were calculated (∆PG‐30; ∆PG‐60; ∆PG‐90).

Blood samples for the analysis (routine high‐performance liquid chromatography HPLC) of plasma norepinephrine (NE, pg/ml) and epinephrine concentrations (E, pg/ml) were drawn at baseline in a supine position and after standing up after completion of the PPH test.

### 2.4 Norepinephrine microdialysis

The experiments were performed in the right ventral thigh 20 cm above knee level. Four microdialysis fibers (DermalDialysis) were inserted intradermally to a length of 1.5 cm by a 25‐gauge cannula. The fibers were placed transversally to the axis of the limb at a distance of approximately 3 cm. Each fiber was perfused with one concentration of norepinephrine (NE) diluted in saline (NE; 10^–5^; 10^–6^; 10^–7^; 10^–8^; flow rate: 4 µl/min). Insertion‐related vasodilatation (neurogenic inflammation) subsides within 60 min (Anderson, Andersson, & Wardell, 1994). Thereafter, NE solution perfusion of the microdialysis membranes was started.

Superficial blood flow was quantified using a laser Doppler imager system (LDI, Moor). LDI scans (256 × 256 pixels, scan resolution 4 pixels/s, distance to skin surface: 50 cm) were recorded at baseline and at intervals of 5 min after NE perfusion commenced. The mean baseline flux value in perfusion units was calculated as the mean from the three acquired baseline pictures. The size of the scanned area was 144 cm^2^. Flux (blood flow intensity) was calculated for each pixel by means of intensity of the Doppler shift of the backscattered laser light (arbitrary perfusion units [PUs]). Intensity of the vasoconstriction was analyzed offline (MLDI 3.0; Moor) separately for each microdialysis fiber. Vasoconstriction expressed in flux values was normalized to baseline (flux value at baseline = 100).

### 2.5 Forehead cooling

A coated ice pack was placed on the participant's forehead for 20 s. This procedure has been demonstrated to reliably cause peripheral vasoconstriction (Muller et al., 2014). Laser Doppler skin blood flow at a single point (Laser Doppler Imager; Moor Instruments Limited) was used to continuously assess acral skin blood flow at the index finger tip (sampling frequency: 20 Hz, time constant: 0.1 s, distance to skin surface: 50 cm). The mean flux value of 30 s was calculated and used as baseline. Acral vasoconstriction was then analyzed for another 20 s during forehead cooling. The relative change in perfusion units was normalized to baseline (flux value at baseline = 100).

### 2.6 Orthostatic hypotension (OH)

Orthostatic hypotension was tested with active standing (Schellong test). Briefly, after lying in a supine position for 15 min, blood pressure and heart rate were measured using a digital sphygmomanometer. The subjects then stood up, and blood pressure and heart rate were recorded directly after getting up as well as after 1, 2, 3, and 5 min. The test was considered pathologic if the SBP decreased ≥ 20 mmHg or the DBP decreased ≥10 mmHg (Freeman et al., 2011).

### 2.7 Skin biopsy and immunochemistry

Skin punch biopsies with a diameter of 5 mm were taken under local anesthesia from the thigh, 20 cm proximal of the patella. The exact procedure has been described previously (Üceyler et al., 2010). The biopsies were fixed with paraformaldehyde 4% and cryo‐protected until use. For the assessment of intraepidermal nerve fiber density (IENFD), 50‐µm sections were cut and immunofluorescence staining with anti‐PGP9.5 (Ultraclone, Isle of Wight, UK, 1:1,000) and Cy3‐conjugated anti‐rabbit IgG (Dianova, Hamburg, Germany; 1:200) as a secondary antibody was performed. IENFD was determined following published counting rules (Lauria et al., 2005). Immunofluorescence double labeling of 20 µm sections was performed to detect P‐α‐synuclein and to quantify autonomic innervation using the following primary antibodies: anti‐phospho‐α‐synuclein (Covance; 1:500) and anti‐PGP9.5 (1:1,000), anti‐TH (Synaptic Systems; 1:100) and appropriate Cy3‐ and Alexa488‐conjugated secondary antibodies (1:200). Six sections per biopsy were assessed first, resulting in two P‐α‐synuclein positive PD cases. We then performed serial sections of all initially negative biopsies by cutting the whole biopsy into 20 µm sections and stained every tenth section with antibodies against P‐α‐synuclein and anti‐PGP9.5 and anti‐tyrosine hydroxylase (TH; sympathetic adrenergic fibers). All sections were analyzed using a fluorescence microscope (Ax10, Zeiss) with CARVII‐system and Visiview software (Visitron GmbH, Puchheim, Germany). The examiner (KD) was blinded for the diagnosis. P‐α‐synuclein deposits were detected by scanning all slides for immunoreactivity of anti‐P‐α‐synuclein within anti‐PGP9.5‐ or anti‐TH‐positive dermal nerve fibers as previously described (Doppler et al., 2014). Sympathetic innervation of pilomotor nerves was quantified by obtaining z‐stacks using the 20x objective of a fluorescence microscope (Ax10, Zeiss) with CARVII‐system (Provitera et al., 2014). The number of fibers/µm crossing a line perpendicular to the axis of the muscle was counted on three optical sections from the top, middle, and bottom, and the average was taken. For quantification of sudomotor and vasomotor innervation, maximum projections of z‐stacks of sweat glands and vessels were obtained at 20x magnification. The percent surface area of the sweat glands and vessels covered with nerve fibers was determined using ImageJ (National Institute of Health, USA).

### 2.8 Sympathetic multiunit microneurography

Multiunit MSNA was acquired with a tungsten microelectrode (0.2 mm wire; 5 µm tip) in the left peroneal nerve posterior of the fibular head as described previously (Sundlof & Wallin, 1977). The nerve activity was referenced to a nearby subcutaneous low‐impedance tungsten electrode, amplified (×50,000), bandpass filtered (700–2,000 Hz), and integrated (time constant 0.1 s). The detection of MSNA and electrocardiogram (ECG) was performed using dedicated software developed in the laboratory of the department of Clinical Neurophysiology, Gothenburg University, Sweden (sampling rates: 200 Hz). Postacquisition signal processing and statistical analyses were also performed using dedicated software from the same institute.

Muscle sympathetic nerve activity bursts were identified automatically and were visually confirmed by the same investigator (GL). Burst frequency (BF; bursts per minute), burst incidence (BI; bursts per 100 heart beats), and burst amplitude (BA) were acquired. Burst frequency represents burst gating and is a useful measure of the degree of activity reaching the effector organs; burst incidence reflects central drive and burst amplitude represents burst strength (Kienbaum, Karlssonn, Sverrisdottir, Elam, & Wallin, 2001). After a successful recording site was established, MSNA and heart rate were recorded for at least 15 min during rest. For analysis of MSNA, the last 5 min of the recording period were used.

In 14 of the included 15 PD patients, a good recording site could be established. In one patient, the experiment had to be terminated since the intramural search took more than 30 min. It is known that extensive intramural searching can be associated with sensory sensations after microneurography.

### 2.9 Statistics

Data were analyzed using the SPSS Statistics (IBM; version 22.0 for Windows) software package. For analysis of the degree of vasoconstriction, repeated‐measures analysis of variances (rm‐ANOVA) was applied using the factors “disease” (PD and healthy controls), “concentration” (NE concentration: 10^–5^; 10^–6^; 10^–7^; 10^–8^) and “time.” For analysis of the OH and forehead cooling, rm‐ANOVA was applied using the factor “disease” (PD and healthy controls). Levene's test was applied to check for homogeneity of variances. Greenhouse‐Geisser correction was applied. Spearman correlation tests were employed to analyze correlations between clinical measures and morphological skin changes. Kolmogorov–Smirnov tests of normality were run for all data sets and parametric or nonparametric statistics were used accordingly. All values are given as medians and interquartile range (IQR) in the case of a non‐normal distribution and as means ± standard error in the case of a normal distribution. Values were considered significant if *p* < .05.

## 3 RESULTS

### 3.1 Resting cardiovascular parameters

At rest, DBP (PD: 85 ± 2.4 mmHg; controls: 75 ± 2.3 mmHg; *p* < .001) was higher in the PD patients while SBP (PD: 137 ± 4.6 mmHg; controls: 131 ± 4.3 mmHg) and HR (PD: 66 ± 3.5 beats/minute; controls: 70 ± 2.6 beats/min; ns) were unchanged.

### 3.2 Postprandial hypotension (PPH)

PPH (drop of SBP ≥ 20 mmHg) occurred in 1 out of 10 healthy volunteers but in 9 of 20 PD patients. The maximum drop in SBP (⊿SBP_max) was higher in PD patients compared to the control group (⊿SBP_max: PD: −21 ± 3.3 mmHg; controls: −9 ± 2.0 mmHg; *t* test; *p* = .029).

Plasma glucose (PG) concentrations did not differ between PD patients and controls (ns; *t* test; PD patients: PG_baseline_: 91.3 ± 2.8; ∆PG‐30:72.0 ± 4.6; ∆PG‐60:77.6 ± 8.1; ∆PG‐90:67.1 ± 9.9; healthy controls: PG_baseline_: 91.9 ± 1.7; PG‐30:84.8 ± 8.8; ∆PG‐60:62.6 ± 11.5; ∆PG‐90:73.0 ± 12.9).

Serum norepinephrine and serum epinephrine were not different between patients and controls (norepinephrine rest: PD: 207.00 (IQR 146.50–324.00) ng/L; controls: 224.50 (IQR 91.50–392.75) ng/L; ns; norepinephrine standing: PD: 471.00 (IQR 200.25–719.00) ng/L; controls: 567.00 (IQR 414.50–891.50); epinephrine rest: PD: 26.00 (IQR 20.00–78.00) ng/L; controls: 20.00 (20.00–32.23); epinephrine standing (PD: 47.65 (IQR 23.90–118.50) ng/L; controls: 35.00 (IQR 24.50–63.05; Mann–Whitney *U* test; ns). In the standing position, serum norepinephrine was higher in both groups compared to supine position (PD: *p* < .001; controls: *p* = .048; Wilcoxon test) while epinephrine was higher only in PD patients (*p* < .001; controls: ns; Wilcoxon test).

### 3.3 Norepinephrine microdialysis

**Figure 1:**
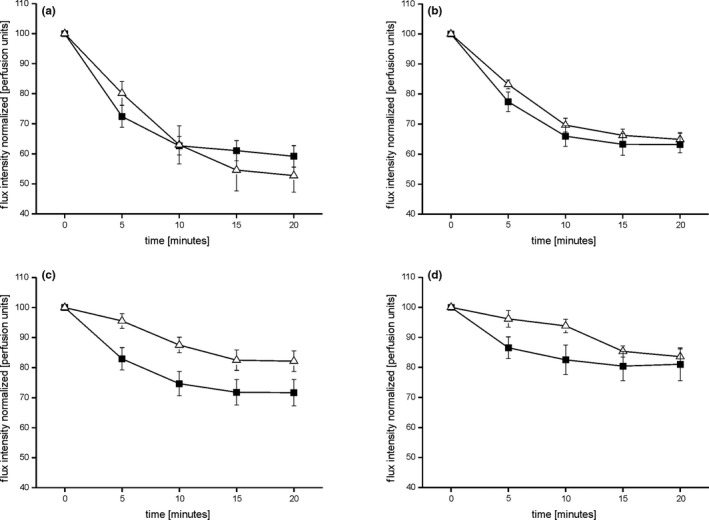
Represents the time course analysis of the degree of vasoconstriction induced by the applied NE concentrations (a: NE 10^–5^; b: NE 10^–6^; c: NE 10^–7^; d: NE 10^–8^) in healthy controls (open triangles) and PD patients (filled squares). The degree of vasoconstriction is represented in flux intensity normalized to baseline in arbitrary perfusion units. The extent of the vasoconstriction did not differ between PD patients and healthy participants (ns; rm‐ANOVA)

As expected, norepinephrine (NE) dose dependently induced vasoconstriction (*F* = 28.786; *p* < .001; rm‐ANOVA; PD: *F* = 4.884; *p* = .004; healthy controls: *F* = 10.854; *p* < .001). The vasoconstriction response did not differ between PD and healthy controls for any of the NE concentrations (10^–5^; 10^–6^; 10^–7^; 10^–8^; ns; rm‐ANOVA; Figure 1).

### 3.4 Forehead cooling

**Figure 2:**
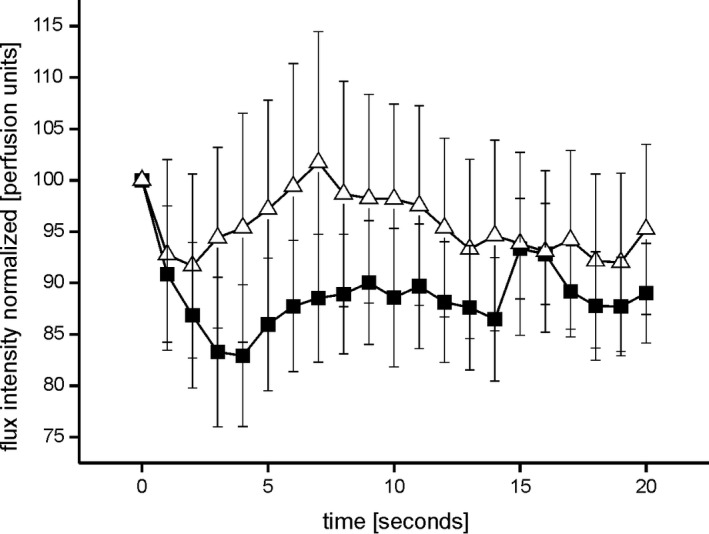
Shows the cold induced acral vasoconstriction over time during forehead cooling in healthy controls (open triangles) and PD patients (filled squares). No differences could be detected between the groups (ns, rm‐ANOVA)

The vasoconstriction response induced by an icepack on the forehead did not differ between healthy controls and PD patients (rm‐ANOVA; ns; Figure 2).

### 3.5 Orthostatic hypotension

**Figure 3:**
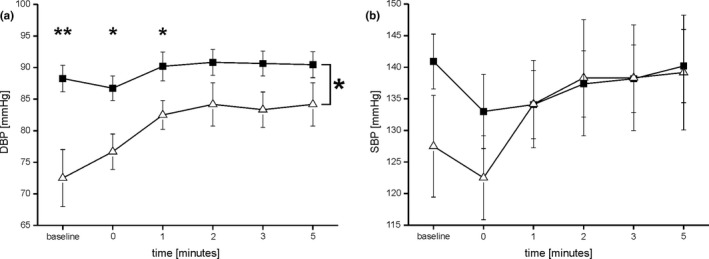
Depicts the diastolic blood pressure (DBP) (a) and the systolic blood pressure (SBP) over time (b) in mmHg during the orthostatic hypotension test. DBP is higher in PD patients (filled squares) compared to healthy controls (open triangles; *F* = 6.533; *p* = .022; rm‐ANOVA). SBP did not differ between PD patients and healthy controls (ns)

Orthostatic hypotension was present in 4 of the 15 PD patients (SBP decreased ≥ 20mmHg: *n* = 2; mean ∆dSP: 26.00 ± 1.41 mmHg; DBP decreased ≥10 mmHg: *n* = 3; mean ∆dBP: 14.33 ± 0.98 mmHg), but in none of the controls. Therefore, the degree of OH is not severe. A quantitative analysis revealed that in this group of PD patients, DBP was higher compared to the control group (*F* = 6.533; *p* = .022; rm‐ANOVA; Figure 3). DBP was higher in the PD patients at baseline (*p* = .004), directly after standing up (*p* = .013) and after one minute of active standing (*p* = .044) compared to the controls. The systolic blood pressure as well as the heart rate did not differ between PD patients and controls (rm‐ANOVA; ns).

### 3.6 Skin biopsies

**Figure 4:**
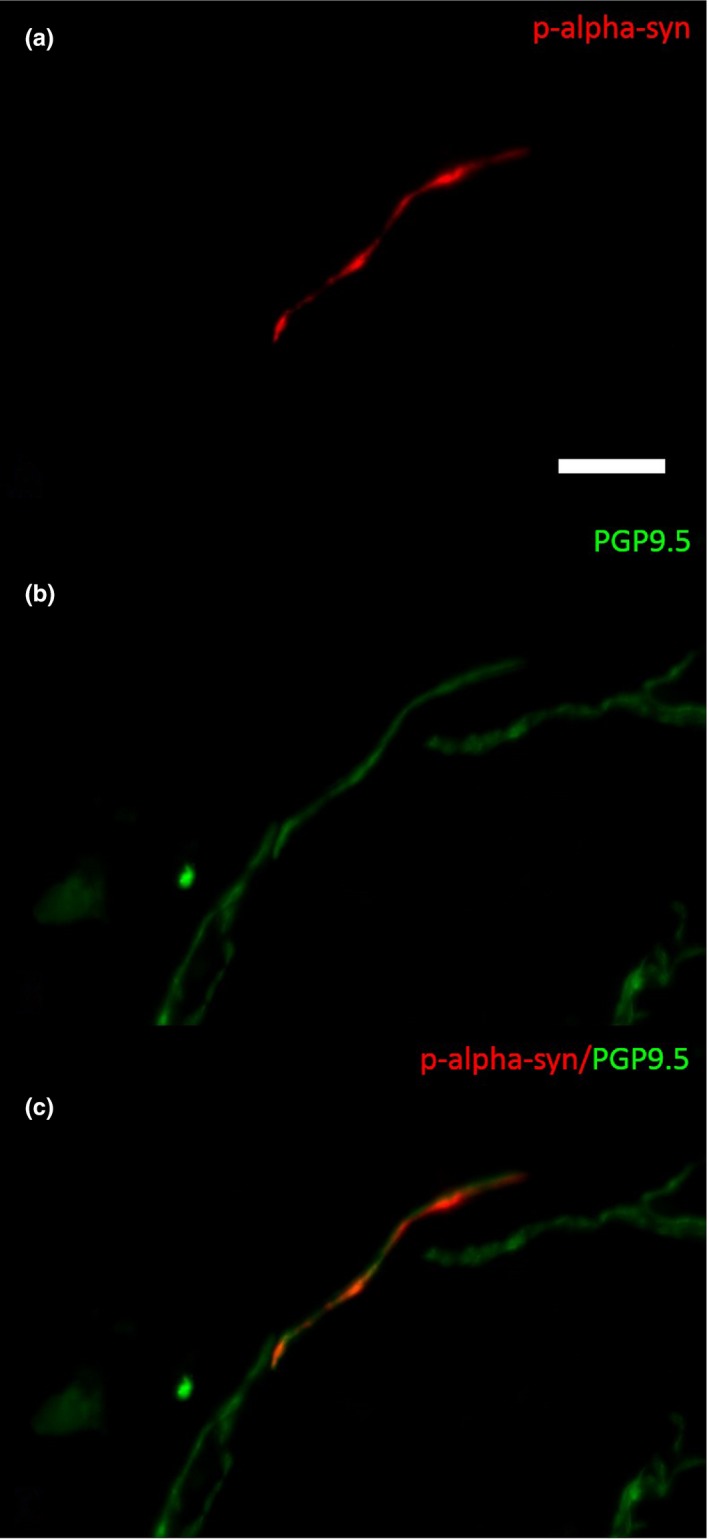
Photomicrograph of a double‐immunofluorescence staining with anti‐α‐synuclein (a, c) and anti‐PGP9.5 (b and c) in a biopsy of a patient with PD. P‐α‐synuclein is detectable within a dermal nerve fiber (c). Bar = 20 µm

Autonomic and somatosensory innervation did not differ between PD patients and controls (Mann–Whitney test; ns). As a secondary finding, P‐α‐synuclein deposits were present in 7 out of 15 PD patients but in none of the healthy participants. These deposits were found in vasoconstrictors (*n* = 4), erector pili muscles (*n* = 2) but also in dermal nerve bundles that were not attached to autonomic structures (*n* = 5). No differences in morphological parameters between PD patients with and without P‐α‐synuclein deposits could be detected (Table 1 and Figure 4).

**Table 1:** Morphological parameters of Parkinson patients and healthy controls

		Parkinson's disease median (IQR) *n* = 15	Controls median (IQR) *n* = 10
Phosphorylated alpha‐synuclein deposits		*n* = 7	*n* = 0
IENFD PGP 9.5 proximal leg [fibers/mm]		6.36 (5.28–9.76)	7.54 (6.55–9.97)
Sweat glands PGP 9.5 [%]		10.40 (6.99–15.27)	9.30 (7.57–11.55)
Erector pili PGP 9.5 [fibers/µm]		0.04 (0.03–0.04)	0.04 (0.02–0.04)
Erector pili TH [fibers/µm]		0.04 (0.03–0.05)	0.03 (0.02–0.04)
Arterial vessel PGP 9.5 [%]		5.20 (4.70–15.37)	7.90 (4.90–11.55)
Arterial vessel TH [%]		7.25 (5.23–11.15)	5.70 (4.15–14.18)

Note

Table shows the morphological parameters of proximal leg skin biopsies in Parkinson patients and healthy controls. No structural differences between the patient and the control group could be found (ns). IENFD, intraepidermal nerve fiber density; TH, tyrosine hydroxylase. %: percent surface area of the sweat glands and vessels covered with immunoreactive nerve fibers.

### 3.7 Sympathetic multiunit microneurography

Burst frequency (BF) at rest was significantly lower in PD patients compared to healthy controls (BF PD: 25.55 ± 0.76 bursts per minute; controls: 31.26 ± 1.38 bursts per minute; *t* test; *p* = .001).

Burst incidence (BI) at rest was significantly lower in PD patients compared to healthy controls (BI PD: 39.06 ± 1.12 bursts per 100 heart beats; controls: 45.21 ± 1.93 bursts per 100 heart beats; *t* test; *p* = .025).

Burst amplitude (BA) did not differ between PD and healthy controls (PD: 0.112 ± 0.018; controls: 0.088 ± 0.012; ns).

### 3.8 Correlations between clinical measures and morphological skin changes in PD

H&Y scale and PD duration but not age correlated negatively with the number of PGP9.5 positive fibers innervating the sweat glands (*r* = −.668; *p* = .007; *r* = −.680; *p* = .005).

The presence of P‐α‐synuclein correlated with PD stage (URPDS III; *r* = .586; *p* = .022) and PD duration (*r* = .537; *p* = .026).

## 4 DISCUSSION

We performed a comprehensive sympathetic assessment of intermediate stage PD patients. We found PPH, OH, and reduced MSNA in the PD patients, indicating impairment of the sympathetic outflow when the autonomic nervous system is challenged. We did not find indications for peripheral sympathetic nerve fiber degeneration by skin biopsy, analysis of circulating catecholamines (see also Groothuis et al., 2011), or adrenoreceptor sensitivity changes in the microdialysis experiments. In our skin samples, P‐α‐synuclein deposits were found in 7 out of 15 PD patients, which was associated with disease duration and severity but not with autonomic dysfunction.

### 4.1 Sympathetic failure in PD

Autonomic failure in idiopathic PD has been described in many other studies (OH: (Donadio et al., 2018); PPH: (Umehara et al., 2014)), and the results of our study support these findings. We found indications for orthostatic and postprandial hypotension and a reduction of MSNA.

There is ongoing controversy whether sympathetic failure in PD is the consequence of a central (e.g., from sympathetic neuron dysfunction in the brainstem (Cersosimo & Benarroch, 2013)) or peripheral (e.g., degeneration of peripheral sympathetic fibers (Dabby et al., 2006)) pathophysiology. This controversy stems from the early occurrence of α‐synuclein in the medullary and pontine autonomic nuclei like the locus coeruleus (Braak et al., 2003; Cersosimo & Benarroch, 2013) but also in the peripheral autonomic nerves (Ikemura et al., 2008). Central pathophysiology seems obvious due to the degenerative processes in PD pathology. However, in particular the results of MIBG uptake in peripheral sympathetic nerves of the heart and the peripheral blood vessels, which occur very early in the PD course (Hirayama et al., 1995) or even in premotor stages (Sakakibara et al., 2019), challenge the assumption of a pure central origin of autonomic impairment in PD. Reduced uptake in MIBG scintigraphy strongly pinpoints toward a degeneration of peripheral sympathetic neurons (Satoh et al., 1999). Moreover, peripheral impairment of sudomotor function measured by QSART could be detected in PD patients (Kawada et al., 2009) showing pathological values even in very early stages (Oh, Lee, Seo, Sohn, & Lee, 2011). Moreover, postmortem analysis revealed a severe degeneration of tyrosine hydroxylase positive noradrenergic peripheral axons in PD (Amino et al., 2005).

The investigation of centrally and peripherally induced sweating in combination offers the possibility to define the site of a sudomotor failure (Low, 2004). Our investigation was comparable. The results (normal serum norepinephrine, normal serum epinephrine, normal TH‐positive nerve fibers in the skin) indicate that in our PD patients peripheral sympathetic failure is unlikely. Moreover, when we circumvented the endogenous release of norepinephrine in the skin by microdialysis, we found no denervation‐related hypersensitivity of peripheral adrenoreceptors on peripheral sympathetic fibers innervating skin blood vessels. Unfortunately, NE microdialysis and MSNA recording was not performed in the same patient group. However, there is no vasoconstriction axon reflex (Schmidt, Weidner, & Schmelz, 2011). That is, P‐α‐synuclein deposits in the skin might not cause autonomic denervation patients which might be responsible for the autonomic failure in our PD. This assumption is supported by the finding that sympathetic function was the same in PD patients with and without P‐α‐synuclein deposits in the skin (Donadio et al., 2014; Doppler et al., 2014). However, our sample size was small and further studies with more patients are needed in the future.

Sympathetic multiunit microneurography has been employed to detect autonomic dysfunction in patients with PD. A previous study investigated a group of patients with moderate to severe PD and detected lower skin sympathetic nerve activity as a measure for arousals (Shindo et al., 2008). In the present study, we investigated muscle sympathetic nerve activity, which is determined by heart rate and blood pressure and the cardiovascular baroreflexes. A recent study employing heart rate variability detected reduced baroreflex sensitivity in drug naïve PD patients (Strano et al., 2016). MSNA is a direct measure of sympathetic activity that is not regulated and influenced by different non‐neuronal cardiovascular reflexes. In contrast to a previous study investigating a heterogeneous group of PD patients (Shindo et al., 2003), we found MSNA to be reduced in our PD patients. The parameter used for the evaluation of sympathetic activity was the burst frequency and burst incidence. The burst amplitude, which reflects burst strength, was unaltered in PD (Kienbaum et al., 2001). The occurrence of MSNA bursts is independent of peripheral sympathetic nerve fiber integrity because MSNA increases with age despite a physiological loss of peripheral nerve fibers (Iwase, Mano, Watanabe, Saito, & Kobayashi, 1991). The medullary circuitry involved in the generation of spontaneous MSNA changes in humans was demonstrated by real time fMRI (Macefield & Henderson, 2010). MSNA and the activation of the nucleus tractus solitarii and the caudal ventrolateral medulla show an inverse relationship (Macefield & Henderson, 2016). In PD, an ascending progression of P‐α‐synuclein positive inclusions has been proposed (Jellinger, 2009) with early deposits within the medulla oblongata (Braak et al., 2006). Therefore, an early impairment of brainstem regions can be suggested. Thus, our results provide evidence for reduced central sympathetic activity in PD. It is speculative, but the significant increase of epinephrine after standing in PD might be a compensatory mechanism to overcome the reduced baroreflex activity.

### 4.2 Increased DBP

In both groups of PD patients, DBP at rest was increased while SBP and heart rate were unchanged. The major reason for diverging results in previous studies showing cardiovascular failure in PD (Wang et al., 2013) might be the moderate severity and the homogeneity of the clinical presentation in our cohorts. Only if PD was advanced, decreased heart rate variability, which would represent a cholinergic not a adrenergic failure could be expected (Wang et al., 2013). In early PD, heart rate is regularly normal (Buob et al., 2010).

How could an increased DBP at rest relate to autonomic impairment as discussed above? The major component of blood pressure regulation is the baroreflex. The efferent branch of the baroreflex is the MSNA, which has been shown to be reduced in our patients. This suggests two explanations. First, contrary to our assumption, the MSNA could be not reduced due to sympathetic failure but as a counter regulation in response to the increased DBP (Sundlof & Wallin, 1977). The increased supine DBP could then reflect impaired baroreflex buffering. The second explanation is the other way round: MSNA is low although DBP is increased. DBP is also controlled by nonsympathetic mechanisms such as local veno‐arteriolar axon reflexes (Henriksen & Sejrsen, 1977), myogenic responses of resistance vessels (Folkow, 1989), or a larger plasma volume (Groothuis et al., 2011), all of which might be itself counter regulations of the impaired sympathetic outflow indicated by decreased MSNA. We cannot prove or disprove one of these assumptions but our findings (no differences in all other tests) are consistent with the second.

### 4.3 Skin biopsies

In the skin biopsies, no differences of TH‐positive nerve fibers between PD patients and controls could be found. Unlike previous investigations (Donadio et al., 2014), we did not find P‐α‐synuclein deposits associated with reduced autonomic innervations. We found P‐α‐synuclein deposits in skin nerve fibers associated with PD duration and severity. These deposits were unrelated to sympathetic function. Another observation was that with advanced PD the innervation of sweat glands with PGP 9.5 positive fibers decreases. We did not assess sweating in our study but the same result was described in a previous study, in which the reduction of sweat gland innervation was not associated with deterioration of sweat gland function which alike heart rate variability represents cholinergic but not adrenergic function (Navarro‐Otano, Casanova‐Molla, Morales, Valls‐Sole, & Tolosa, 2015).

### 4.4 Limitations of the study

One limitation of our study is that not all tests could be performed in all subjects. Two different patients and control groups were investigated because of the exhausting protocol, and because the two centers which participated in our study have different methods to assess sympathetic adrenergic activity. Therefore, the samples were splitted (15 and 20 patients). Since the biographical data of the patients of the two investigated groups did not differ, we feel that our assumptions are nevertheless justified. Another key limitation of our conclusion might represent the lower sensitivity of peripherally mediated functional vasoconstriction tests. We did not detect any denervation‐related hypersensitivity of peripheral adrenoreceptors on peripheral sympathetic fibers innervating skin blood vessels by norepinephrine microdialysis. There is no evidence for vasoconstriction axon reflex. However, our results showing the clear reduction of MSNA in PD patients pinpoints toward a dominant role of centrally altered autonomic drive. Unfortunately, the routine investigations MIBG scintigraphy and head tilt test were not performed in our study. However, since we use multiple other tests we feel that our results are still valid. Another major limitation is that the study was performed without withdrawing medication. This could have a major impact on our OH and PPH findings since dopaminergic medication could be associated with a drop in blood pressure (Calne, Brennan, Spiers, & Stern, 1970; Noack, Schroeder, Heusser, & Lipp, 2014). However, an adequate wash out which means discontinuation of the medication for two or more days (Harder, Baas, Bergemann, Demisch, & Rietbrock, 1995) seemed not to be justified for the purpose of the study.

## 5 CONCLUSION

We provide evidence for functional rather than structural efferent sympathetic changes in intermediate PD patients. Our data with reduced MSNA argue in favor of central sympathetic impairment while we found no indications for peripheral sympathetic nerve fiber degeneration or dysfunction. However, longitudinal studies including treatment naïve PD patients are needed to further elucidate the nature and progression of autonomic pathology in PD.

## CONFLICT OF INTEREST

The authors have no conflict of interest to report.

## Data Availability

The data that support the findings of this study are available from the corresponding author upon reasonable request.
